# The Protective Effect of *Marsdenia tenacissima* against Cisplatin-Induced Nephrotoxicity Mediated by Inhibiting Oxidative Stress, Inflammation, and Apoptosis

**DOI:** 10.3390/molecules28227582

**Published:** 2023-11-14

**Authors:** Zhiguang Zhang, Boya Liang, Wugemo Jike, Runtian Li, Xinxin Su, Jie Yu, Tongxiang Liu

**Affiliations:** 1 School of Pharmacy, Minzu University of China, Beijing 100081, China; 20400226@muc.edu.cn (Z.Z.); 22301906@muc.edu.cn (B.L.); 22301883@muc.edu.cn (W.J.); lrt870229@163.com (R.L.); suxinxin0331@163.com (X.S.); 21301857@muc.edu.cn (J.Y.); 2 Key Laboratory of Ethnomedicine, Ministry of Education, Minzu University of China, Beijing 100081, China

**Keywords:** *Marsdenia tenacissima*, cisplatin, nephrotoxicity, oxidative stress, inflammation, apoptosis

## Abstract

Cisplatin (Cis) is considered to be one of the most effective drugs for killing cancer cells and remains a first-line chemotherapeutic agent. However, Cis’s multiple toxicities (especially nephrotoxicity) have limited its clinical use. *Marsdenia tenacissima* (Roxb.) Wight et Arn. (MT), a traditional Chinese medicine (TCM) employed extensively in China, not only enhances the antitumor effect in combination with Cis, but is also used for its detoxifying effect, as it reduces the toxic side effects of chemotherapy drugs. The aim of this study was to explore the therapeutic effect of MT on Cis-induced nephrotoxicity, along with its underlying mechanisms. In this study, liquid–mass spectrometry was performed to identify the complex composition of the extracts of MT. In addition, we measured the renal function, antioxidant enzymes, and inflammatory cytokines in mice with Cis-induced nephrotoxicity and conducted renal histology evaluations to assess renal injury. The expressions of the proteins related to antioxidant, anti-inflammatory, and apoptotic markers in renal tissues was detected by Western blotting (WB). MT treatment improved the renal function, decreased the mRNA expression of the inflammatory factors, and increased the antioxidant enzyme activity in mice. A better renal histology was observed after MT treatment. Further, MT inhibited the expression of the phospho-NFκB p65 protein/NFκB p65 protein (p-p65)/p65, phospho-inhibitor of nuclear factor kappa B kinase beta subunit/inhibitor of nuclear factor kappa B kinase beta subunit (p-IKKβ/IKKβ), Bcl-2-associated X (Bax), and Cleaved Caspase 3/Caspase 3 proteins, while the expression of nuclear factor-erythroid 2-related factor 2 (Nrf2), heme oxygenase-1 (HO-1), Recombinant NADH Dehydrogenase, Quinone 1 (NQO1), and B-cell lymphoma-2 (Bcl-2) was increased. The present study showed that MT ameliorated renal injury, which mainly occurs through the regulation of the Nrf2 pathway, the NF-κB pathway, and the suppression of renal tissue apoptosis. It also suggests that MT can be used as an adjuvant to mitigate the nephrotoxicity of Cis chemotherapy.

## 1. Introduction

With the development of medical technology, intensive research has brought about advances in cancer treatment, but chemotherapeutic agents still remain the foundation of systemic treatment for many cancers [1]. Platinum-based compounds are important anticancer chemotherapeutic agents [2]. Cis was first licensed for use in clinical practice in 1978, approved for a wide range of pediatric and adult malignant solid tumors, such as esophageal, testicular, breast, cervical, and small-cell lung cancers [3]. Considered one of the best potent chemotherapy drugs, Cis remains the first-line agent in cancer therapy [4]. However, the clinical use of Cis is closely associated with many adverse effects, with Cis-induced nephrotoxicity being a common contributor to acute kidney injury (AKI) [5]. Cis nephrotoxicity involves multiple mechanisms. The accumulation of platinum in renal tissues induces cytotoxicity in the kidney, as drug transporters in the basement membrane increase the uptake of drugs in the kidney [6,7]. The accumulation of Cis triggers the elevated expression of pro-inflammatory cytokines, e.g., tumor necrosis factor-α (TNF-α), interleukin-6 (IL-6), and interleukin-1β (IL-1β), which, along with the massive production of ROS and the disruption of antioxidant defenses, leads to the activation of the Keap1/Nrf2 pathway. Nrf2 is a key transcription factor in combating oxidative stress and initiating redox pathways. Its activation increases the activity of antioxidant enzymes such as superoxide dismutase (SOD), glutathione peroxidase (GSH-Px), and catalase (CAT) and also induces the expression of downstream target genes such as HO-1 and NQO1 by activating antioxidant response elements. Thus, it exerts antioxidant effects, reduces cellular oxidative stress, improves renal function, and attenuates renal injury [8,9,10,11]. In addition, Cis selectively damages the proximal tubule S3 segment cells, manifesting as necrosis and apoptosis [12,13]. Together, the pronounced inflammatory response and damage to the renal vascular system cause vasoconstriction, reduced blood flow, and ischemic injury. Such abnormalities can contribute to AKI. As mentioned previously, the pathological mechanisms of AKI are complex, and it is difficult to achieve the desired therapeutic effect via the application of single-target and synthetic drugs. There is an urgent need to develop safe and potent agents to protect against or treat kidney injury. 

MT was first described in *Dian Nan Ben Cao* [14]. As a traditional Dai medicine, MT is also known as Dai-Bai-Jie and Ya-Jie-Xian-Da, which means: medicine for all poisons [15]. It has been extensively employed to treat food poisoning, painful urination, sore throat, and stomach pain [15,16]. The extracts of MT are prepared into injections and tablets. It has been marketed in China under the trade name of “Xiao ai ping” (XAP). Clinically, it is mainly used as an adjuvant treatment for chemotherapy, which can effectively inhibit cancer growth and reduce the occurrence of various side effects from chemotherapy. Several studies have confirmed that oral or injectable XAP can reduce the adverse reactions after chemotherapy. The occurrence of adverse effects including bone marrow suppression, impairment of liver and kidney function, nausea, diarrhea, and constipation was significantly lower in the advanced gastric cancer group treated with XAP in combination with chemotherapy compared to that in the control group [17]. A meta-analysis showed that XAP combined with platinum chemotherapy exhibited stronger anti-cancer effects, reduced adverse events, and allowed for a better quality of life [18]. In vitro activity experiments showed that MT can effectively scavenge free radicals of DPPH, -OH, and ABTS, and inhibit the production of NO, TNF-α, IL-1β, and IL-6 in LSP-induced macrophages, thus achieving a detoxification effect [19]. MT’s main chemical components are divided into two major groups: organic acids, represented by chlorogenic acid (CA); and C-21 steroids, represented by tenacissoside H (TH). Pharmacological experiments have demonstrated that organic acid components such as CA, shikimic acid, and protocatechuic acid have nephroprotective effects on Cis-induced AKI [20,21,22]. In addition, TH in MT exerted anti-inflammatory effects through the modulation of the Nf-κb and p38 pathways in zebrafish [23]. The total saponins of MT attenuated CCl4-induced and paracetamol-induced liver injury [24,25]. MT and its compound dresgenin were protective against Adriamycin-induced cardiotoxicity in mice in vivo [26].

Based on the clinical application and pharmacological studies regarding MT, in this study, we investigated the effects of MT on the histology, oxidative stress, apoptosis, and inflammation subsequent to Cis induction. We evaluated the underlying ameliorative mechanism of action of MT on Cis-induced kidney toxicity in mouse models. Significantly, this study is important in that it describes, for the first time, a potential mechanism of MT nephroprotection.

## 2. Results

### 2.1. Chemical Composition of MT Identified by UPLC-Q/TOF-MS 

**Figure 1:**
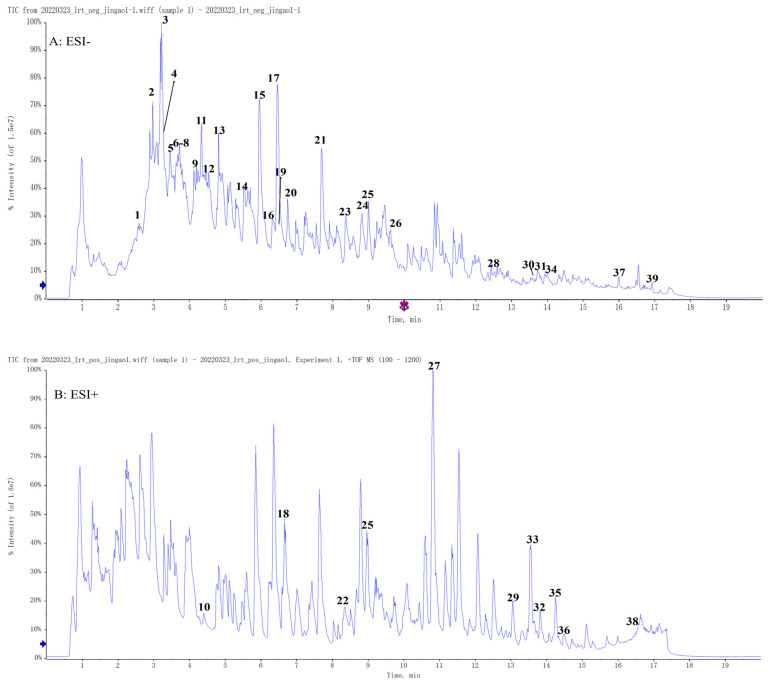
UPLC-QTOF-based TIC curves in positive and negative ion modes: (**A**) negative ion mode; (**B**) positive ion mode. The numbers labeled in the figure represent the order and retention times of the compounds in Table 1.

The typical total ion chromatography (TIC) (positive, negative) is shown in Figure 1. The composition was defined by comparison using database search software (PeakView 1.2), employing the MS exact mass and the MS/MS spectral fragmentation referenced in the literature. Through comparison of their theoretical value, all the identified components showed a quality precision of less than 10 ppm. Table 1 reveals the details of 39 components; the chemical composition of MT is mainly organic acids, flavonoids, and C-21 steroids. The peak value of the molecular ion of TH in positive ion mode is *m*/*z* 817.4377 [M + Na]^+^. The cleaved ionic fragments are *m*/*z* 757, *m*/*z* 655, *m*/*z* 633, *m*/*z* 311, and *m*/*z* 293. In the positive ion mode, the molecular ion peak of CA is *m*/*z* 353.0918 [M + H]^+^. The cleaved ion fragments are *m*/*z* 191, *m*/*z* 179, *m*/*z* 161, *m*/*z* 127, and *m*/*z* 85. 

### 2.2. MT Ameliorates Cis-Induced Kidney Damage in Mice

**Figure 2:**
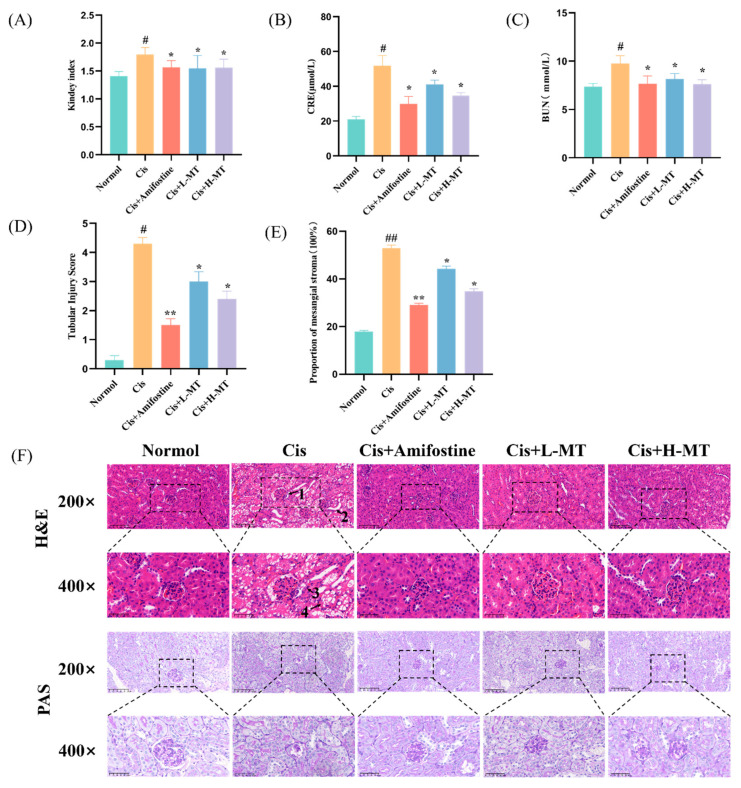
MT improved kidney injury in mice that were exposed to Cis: (**A**) kidney indices for mice; (**B**) CRE levels in mice serum; (**C**) BUN levels in mice serum; (**D**) tubular injury score; (**E**) proportion of mesangial stroma (100%); (**F**) HE dyeing of kidney (original enlargement 200×, top row; partial enlargement image 400×, bottom row). PAS for staining of kidney slices (original enlargement 200×, top row; partial enlargement image 400×, bottom row): 1. glomerular shrinkage; 2. tubular dilation; 3. infiltration of the kidney with inflammatory cells in the interstitium; 4. detached renal tubular epithelial cells. The results are presented as the mean ± standard deviation (SD) (*n* = 8). * *p* < 0.05 vs. Cis; ** *p* < 0.01 vs. Cis; # *p* < 0.05 vs. one-way ANOVA for vehicle control; ## *p* < 0.01 vs. one-way ANOVA for vehicle control.

As shown in Figure 2A, the renal index was found to be significantly higher in the Cis group, but this notably decreased after treatment with amifostine or MT. Furthermore, serum CRE and BUN levels were checked as indicators of kidney function. The rise in CRE and BUN levels in the Cis group suggests renal filtration dysfunction. Kidney function was, in part, improved in Cis-induced kidney damaged mice by the application of oral MT treatment (*p* < 0.05) (Figure 2B,C).

### 2.3. Histopathological Study 

Kidney tissues were obtained using HE and PAS staining to further assess the extent of Cis-induced kidney injury (Figure 2D–F). The kidneys of the control mice did not exhibit any notable pathological alterations. The results of the HE dye indicated that the kidney tissue in the Cis group showed significant cellular necrosis and inflammatory infiltration, while a significant improvement in the lesions, such as inflammatory cell infiltration and glomerular atrophy, was observed after MT intervention, especially in the high-dose group (*p* < 0.05). In addition, in PAS staining, in the Cis group, the kidney tubules exhibited a significant accumulation of glycogen, while the MT intervention significantly improved the tubular cell necrosis and glycogen deposition (*p* < 0.05). These findings suggest that MT exerts a preventative impact on the kidney in terms of pathology. 

### 2.4. Effect of MT Treatment on Oxidative Stress Parameters

**Figure 3:**
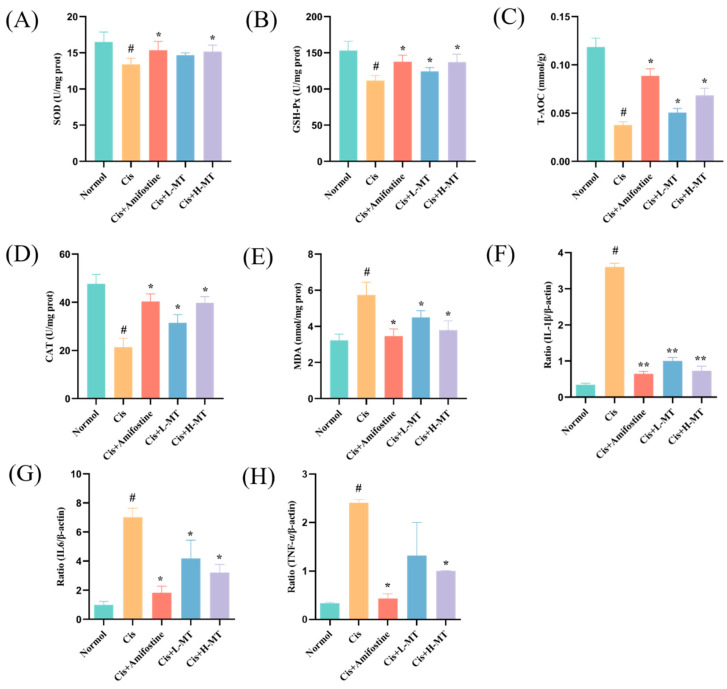
The influence of MT on antioxidant capacity and mRNA levels of pro-inflammatory cytokines in the renal tissue: (**A**) Kidney SOD; (**B**) GSH-Px; (**C**) T-AOC; (**D**) CAT; (**E**) MDA; (**F**) IL-1β; (**G**) IL-6; (**H**) TNF-α. Data are presented as the mean ± standard deviation (SD) (*n* = 8). * *p* < 0.05 vs. Cis; ** *p* < 0.05 vs. Cis; # *p* < 0.05 vs. vehicle control by one-way ANOVA.

The content of MDA is an indirect indicator of the levels of lipid peroxidation and cellular injury, whereas SOD, CAT, GSH-Px, and T-AOC are all types of antioxidants. To explore the antioxidant effect of MT on mice, we measured the activities of peroxidases (SOD, GSH-Px, CAT, and T-AOC), as well as the MDA content. As shown in Figure 3A–E, the above mentioned oxidative factor activities were greatly reduced (*p* < 0.05), and the MDA level was markedly enhanced (*p* < 0.05) in the Cis group compared to those in the control mice. This shows that Cis may lead to an imbalance in the renal oxidative system. Intervention with MT or amifostine resulted in a marked improvement in the CAT, T-AOC, GSH-Px, and SOD activities (*p* < 0.05) and a reduction in the MDA level (*p* < 0.05). The findings showed that MT alleviated Cis-induced AKI by reducing peroxide production and inhibiting lipid peroxidation reactions.

### 2.5. Effect of MT on Inflammation-Related Gene Expression in Renal Tissues

The anti-inflammatory effects of MT were investigated by detecting the levels of relevant inflammatory genes using RT-PCR. As shown in Figure 3F–G, the levels of the inflammatory factors (IL-1β, IL-6, and TNF-α) in the model group were significantly higher than those in control mice (*p* < 0.05), suggesting that renal injury in the model group may have led to the release of inflammatory factors. In contrast, the mRNA expression of inflammatory factors such as IL-1β, IL-6, and TNF-α was significantly reduced in the treated group (*p* < 0.05). This suggests that, after treatment with MT and amifostine, the release of inflammatory factors may be reduced, thus ameliorating Cis-induced renal injury.

### 2.6. MT Activates the Nrf2-Mediated Antioxidant Response

**Figure 4:**
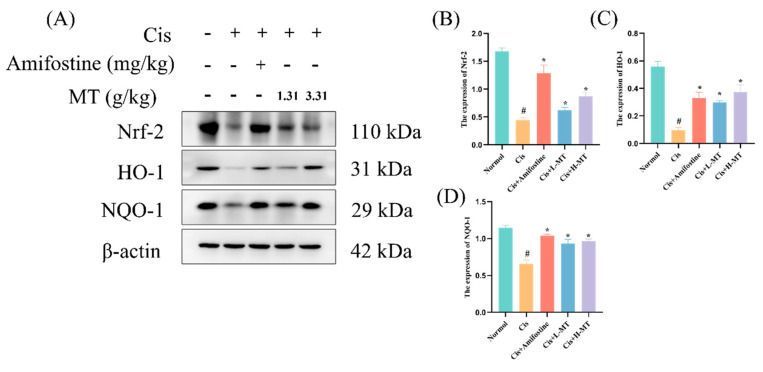
The activation of the Nrf2-mediated antioxidant response was initiated by MT. (**A**) The expression of Nrf2, NQO1, and HO-1 was detected in the kidney by Western blot analysis; (**B**) Nrf2 protein expression; (**C**) HO-1 protein expression; and (**D**) NQO1 protein expression. Data are presented as the mean ± standard deviation (SD) (*n* = 3). * *p* < 0.05 vs. Cis; # *p* < 0.05 vs. vehicle control by one-way ANOVA.

**Figure 5:**
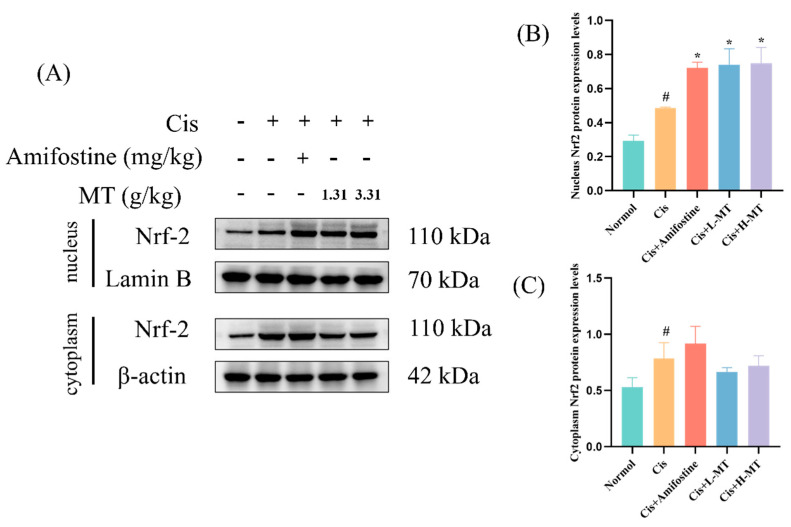
Nrf2 protein was expressed in the nucleus and cytoplasm after MT intervention. (**A**) The expression of Nrf2 in the nucleus and cytoplasm of the kidney was detected by Western blot analysis; (**B**) nucleus Nrf2 protein expression levels; and (**C**) cytoplasm Nrf2 protein expression levels. Data are presented as mean ± standard deviation (SD) (*n* = 3). * *p <* 0.05 vs. Cis; *# p <* 0.05 vs. vehicle control by One-way ANOVA.

Oxidative stress is an important factor causing the development of kidney injury, in which Nrf2 is an antioxidant transcription factor that protects cells from oxidative stress by regulating the transcription of downstream antioxidant genes such as HO-1 and NQO1. Therefore, we explored the mechanism by which MT protected against oxidative stress via WB detection of the expression of Nrf2 and its downstream proteins. In Figure 4, it is obvious that the expression levels of Nrf2, NQO1, and HO-1 in the kidney were reduced in the Cis group. This is an indication that the Nrf2 signaling pathway was notably repressed in the Cis-induced AKI. Interestingly, after amifostine or MT treatment, the expression of the above proteins was clearly higher than that in the Cis group. Meanwhile, we further determined the protein expression of Nrf2 upon entry into the nucleus, which was shown to be similar to the total Nrf2 protein expression (Figure 5). The findings suggest that MT, which may attenuate oxidative stress by modulating the Nrf2/HO-1 signaling pathway, can alleviate Cis-induced kidney injury in mice (as detailed in Supplementary Materials Figure S1).

### 2.7. MT Inhibits the Activation of NF-κB in Mice

**Figure 6:**
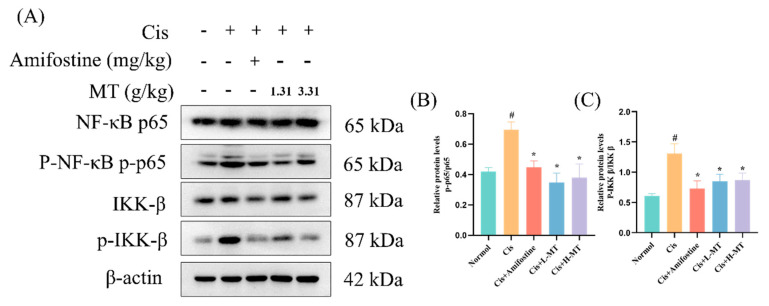
MT suppresses the NF-κB pathway in cis-induced nephrotoxic mice. (**A**) WB analysis of the expression of p65, p-p65; IKK β and p-IKK β in the kidney; (**B**) p-p65/p65 protein expression; and (**C**) p-IKK β/IKK β protein expression. Data are presented as the mean ± standard deviation (SD) (*n* = 3). * *p* < 0.05 vs. Cis; # *p* < 0.05 vs. vehicle control by one-way ANOVA.

p65 has been involved in the regulation of inflammatory cytokine production. In this study, the analysis of p-p65and p-IKKβ in kidney tissues was detected by WB. The manifestation of p-p65 and p-IKKβ was markedly increased in the kidney tissues of Cis-treated mice compared with normal mice. p-p65 and p-IKKβ were significantly attenuated in renal tissues after Amifostine or MT interventions in contrast to the Cis group (Figure 6). 

### 2.8. MT Attenuates Cis-Induced Apoptosis

**Figure 7:**
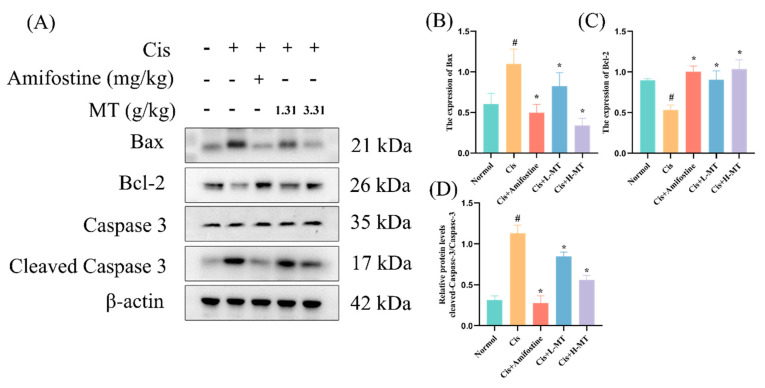
MT decreased apoptosis in Cis-induced nephrotoxic mice. (**A**) WB analysis of the expression of Bax, Bcl-2, caspase 3, and cleaved caspase 3 in the kidney; (**B**) Bax protein expression; (**C**) Bcl-2 protein expression; and (**D**) cleaved caspase 3/caspase 3 protein expression. Data are presented as mean ± standard deviation (SD) (*n* = 3). * *p* < 0.05 vs. Cis; # *p* < 0.05 vs. vehicle control by one-way ANOVA.

Renal apoptosis is associated with decreased caspase 3 activity, but Cis increases caspase 3 activity. To confirm that MT inhibits Cis from inducing apoptosis in kidney injury, we measured the levels of Bax, Bcl-2, caspase 3, and cleaved caspase 3 in kidney tissues using the WB method. The results showed that, in the model group, the expression levels of Bax and cleaved caspase 3 were significantly increased, and the expression level of Bcl-2 was significantly decreased compared with those noted in the control group (Figure 7). These changes were significantly improved after amifostine and MT pretreatment. The above results indicated that MT had a significant inhibitory effect on Cis-induced apoptosis in mouse kidney tissues.

## 3. Discussion

Cis, which is an excellent and potent antitumor agent, is allowed only restricted clinical use due to its serious side effects, in particular, kidney toxicity [27]. Its nephrotoxicity is due to a rapid decrease in renal excretion, which augments the concentration of protein metabolites (BUN and CRE) [27,28,29]. The potential pathology of Cis-induced nephrotoxicity includes renal tubular damage, oxidative stress, apoptosis, and inflammation. [30]. At present, there are no valid drugs or methods for treating the kidney damage caused by Cis [3]. MT is a Dai medicine that is widely used to treat food poisoning, painful urination, sore throat, and stomach pain [14]. Today, XAP (extract of MT) is extensively employed in China, not only in clinical practice for various tumors, but also for clinical adjuvant chemotherapy to reduce the toxicity of chemotherapeutic drugs [17,31]. Previous literature has reported that MT is rich in organic acid components and C-21 steroidal components, which are chemical factors with strong antioxidant, anti-inflammatory effects [20,23]. In this study, a Cis-induced AKI model was developed to assess MT’s effects. Experiments confirmed that MT is indeed an antioxidant and can inhibit inflammation and apoptosis to reduce Cis-induced renal toxicity. 

In this research, 39 compounds from MT were identified using UPLC-Q/TOF-MS. Among these were 11 organic acids, 7 flavonoids, 13 C-21 steroids, 5 terpenoids, and 3 coumarins. It was reported in the literature that organic acid components (CA, caffeic acid, etc.) could counteract Cis-induced nephrogenic injury; similarly, flavonoids (quercetin, apigenin, wogonin, etc.) had nephroprotective effects [32,33,34]. C-21 steroids (TH and dresgenin) have significant anti-inflammatory and antioxidant effects, as do terpenoids (oleanolic acid and betulinic acid) [23,26,35,36]. It was found that MT treatment was effective in alleviating Cis-induced renal damage through diminishing oxidative stress and inflammation.

The levels of CRE and BUN in the blood are widely used as the main indicators to assess renal function [37,38]. In this work, we showed that Cis can cause serious renal toxicity in mice, with significant increases in serum CRE and BUN vales and a decrease in body weight, producing swollen, congested, and hypertrophied kidneys, leading to an increase in the renal index. When mice were administered amifostine or MT, the Cis-induced decrease in kidney function and structural kidney damage was ameliorated, with the amifostine and MT high-dose groups showing a more pronounced improvement, resulting in a marked decline in the CRE and BUN levels and a significant reduction in pathological kidney damage.

Previous studies have elucidated that oxidative stress is a key player in Cis-induced AKI and that, in the body, a dynamic balance between ROS production and clearance is maintained to preserve homeostasis [39,40]. Cis disrupts this balance by overproducing ROS and compromising the antioxidant defense system. This results in the decreased generation of critical antioxidants, i.e., SOD, T-AOC, and CAT [10,41]. Due to the reduced antioxidant capacity, oxidative stress is increased after Cis intervention [3]. High ROS production causes lipid peroxidation, which results in a large amount of MDA (a marker of lipid peroxidation) [42]. Many other authors have also reported an increase in MDA levels after Cis administration [43]. In this work, we analyzed the levels of relevant antioxidant enzymes affected by Cis-induced kidney toxicity. The results showed that the levels of different antioxidant factors (SOD, CAT, GSH-Px, and T-AOC) were markedly lower in the model group when contrasted with those of the control mice; the MDA level was dramatically higher, indicating an increase in lipid peroxidation. Interestingly, amifostine or MT pretreatment improved the levels of antioxidant factors and MDA. These results suggest that MT can effectively alleviate Cis-induced nephrotoxicity in mice by enhancing antioxidant ability and lowering excess lipid peroxidation. Nrf2 is one of the most important transcription factors in the cellular oxidative stress pathway, capable of upregulating the expression levels of various antioxidant proteins and phase II detoxification enzymes in the organism, as well as scavenging free radicals generated by oxidative stress in vivo [44]. Nrf2 is mainly found in the cytoplasm, where it binds to Keap1 and is in an inactive state. When the cell is stimulated by oxidants and other oxidative stress, Nrf2 is activated via its uncoupling from Keap1 and undergoes transmembrane transport into the nucleus, which initiates the expression of antioxidant enzymes downstream of the Nrf2-ARE signaling pathway, playing an antioxidant role [45]. Notably, there was downregulation of Nrf2, HO-1 and NQO1 expression in the Cis group, but amifostine or MT treatment significantly reversed the downregulation of these proteins. As the activated Nrf2 protein enters the nucleus to form a heterodimer with the sMaf protein, it binds to the ARE, initiates the expression of a variety of downstream cytoprotective genes, and participates in biological processes such as the scavenging of ROS, the regulation of autophagy, and the repair of damaged cells [44,46]. Therefore, we further determined the expression of Nrf2 protein in the cytoplasm and nucleus. The results were similar to those for the total Nrf2 protein expression, which were significantly higher in the treated group compared with the model group. These results suggest that the chemical constituents in MT may enhance antioxidant capacity and alleviate renal injury through activation of the Nrf2 pathway.

Nephrotoxicity induced by Cis is related to inflammation. TNF-α levels were increased in mice with Cis-induced renal toxicity, and TNF-α inhibition or knockdown significantly alleviated Cis-induced renal insufficiency and injury, suggesting that heightened TNF-α production is a key factor in Cis-induced AKI [47]. Studies have shown that TNF-α can stimulate the production of ROS, which can activate NF-κB, which in turn induces the production of inflammatory agents (e.g., TNF-α, IL-1β, and IL-6), further aggravating inflammation [48]. TH, a quality control marker for MT, further exacerbates the inflammatory response by regulating NF-κB to modulate inflammatory factors (e.g., IL-1β, IL-6), thereby inhibiting the LPS-induced inflammatory response [23]. Quercetin, a compound with antitumor, anti-inflammatory, and antioxidant effects, was shown by real-time PCR to downregulate the mRNA expression of IL-1, IL-6, and TNF-α compared to that in the Cis-treated mice [32]. Similar to these findings, MT administration exhibited significant downregulation effects on the mRNA levels of TNF-α, IL-1β, and IL-6 in mouse kidneys. It is clear that NF-κB is a key transcription factor implicated in the regulation of inflammation [49]. Pro-inflammatory cytokines activate the IKK complex (IKKβ, IKKα, and NEMO), which phosphorylates the IκB protein leading, to its proteasomal degradation and the release of the NF-κB/Rel complex. The active NF-κB/Rel complex is activated by phosphorylation and translocated into the nucleus, where it induces target gene expression, alone or in combination with other transcription factors [50]. Thus, activation of p-IKKβ and p-p65 essentially determines the activation of the NF-κB pathway. In this study, we found that the activation of p-p65 and p-IKKβ was dramatically suppressed after amphotericin and MT treatment, compared with that in the Cis group, according to WB experiments. Some of the discoveries provide support for our findings that MT may have anti-inflammatory effects.

Apoptosis is considered to be another key factor in Cis-induced nephrotoxicity [51,52]. Bax and Bcl-2 are two essential players in the Bcl-2 family of proteins involved in renal tubular apoptosis [53]. Previous research has also reported that Cis triggers the intrinsic apoptotic pathway via caspase 3 and caspase 9 [54]. In this project, the effects of amifostine and MT on AKI were assessed by examining the levels of caspase 3, cleaved-caspase 3, Bax, and Bcl-2. The results showed that, after amifostine and MT interventions, Bcl-2 was significantly increased, and the expression of Bax and Cleaved-caspase 3/caspase 3 was decreased, compared with those noted in the Cis group. In conclusion, MT remarkably decreased apoptosis in renal tissues and exerted good renoprotective effects in Cis-induced AKI in mice.

## 4. Materials and Methods

### 4.1. Materials

#### 4.1.1. Reagents and Antibodies

Methanol and acetonitrile were obtained from Thermo Fisher Scientific (Waltham, MA, USA). Shanghai Aladdin Biochemical Technology Co., Ltd. provided HPLC-grade formic acid (Shanghai, China). Tongguang Fine Chemical Co., Ltd. (Beijing, China) provided the other analytical grade reagents. Obtaining ultrapure water was possible, thanks to Wowhaha Co., Ltd. (Hangzhou, China).

Cis was provided for injection (lyophilized form) (SFDA approval number: H20023460, batch number: 1B0096B02) from Qilu Pharmaceutical Co., Ltd. (Shandong, China). Amifostine (batch number: C13744263) was provided from Solabao Technology Co., Ltd. (Beijing, China). TH and CA were obtained from the National Institutes for Food and Drug Control (batch number: 111913-201803; 110753-202119, respectively). Macklin Biochemical Co., Ltd. (Macklin, Shanghai, China) provided (2-Hydroxypropyl)-β-cyclodextrin. SOD (batch number:20220704), creatinine (CRE) (batch number: 20220706), total antioxidant capacity (T-AOC) (batch number: 20220705), blood urea nitrogen (BUN) (batch number: 20220629), GSH-Px (batch number: 20220705), CAT (batch number: 20220704), and malondialdehyde (MDA) (batch number: 20220705) were obtained from Nanjing Jiancheng Bioengineering Institute (Nanjing Jiancheng Bioengineering Institute, Nanjing, China). Star script IIRT Mix with the gDNA Remove kit (Lot#CGE121) were acquired from GenStar Biotech (GenStar, Beijing, China). LabLead Biotechnology supplied the BCA Protein Assay kit (Lot: 5000052021) (LabLead, Beijing, China). Anti-p65 (Lot: BB10125523), anti-Bax (Lot: BA12063356), anti-Nrf2 (Lot: BB01286971), anti-Bcl-2 (Lot: BB09268767), anti-HO-1 (Lot: BB07252595), anti-NQO-1 (Lot: BB10121621), and anti-caspase-3 (Lot: BA08247137) antibodies were procured from Bioss Biotechnology (Bioss, Beijing, China). Anti-cleaved caspase-3 (Lot: ab2302), anti-p-p65 (Lot: ab86299), anti-IKKβ (Lot: ab124957), and anti-p-IKKB (Lot: ab59195) were purchased from Abcam Plc. (Abcam, Cambridge, UK). Primary antibody dilution (Lot: P0256) was purchased from Shanghai Beyotime Biotechnology Co (Beyotime, Shanghai, China). The antibody sources and their dilution ratios are shown in Table 2.

#### 4.1.2. Plant Material 

The MT stems were collected in September 2021 from Tangli Mountain, China, and validated by Kunming Plantwise Biotech Co., Ltd. (Kunming, China). The specimen (Number: MT-202109) was kept at Minzu University of China (Beijing, China).

### 4.2. Methods

#### 4.2.1. Preparation of MT Aqueous Extract

The dried stems of MT (500 g) were dipped into 70% ethanol (1:10, *w*/*v*) for 0.5 h, with condensation reflux abstraction for 1.5 h. After filtering through two tiers of muslin cloth, the raw drug remnant was gathered and subsequently poached in 70% ethanol at a liquor ratio of 1:8 (*w*/*v*) for 1 h at condensation reflux. Using a solid–liquid ratio of 1:8 for 1 h, raw drug residue was collected and extracted again with 70% ethanol. After combining the three extraction solutions, they were reduced-pressure concentrated. The extracts were dried in a lyophilizer and stored in a desiccator. Before use, solubilizing was performed on the dried extracts in distilled water to the indicated concentrations. 

#### 4.2.2. Sample Preparation

A total of 1 mg of the above dried MT extract was weighed precisely, dissolved in 1 mL of chromatographic methanol, permeated through a 0.22 μm filter tip, and the filtered solution was deposited into a sample bottle to await testing.

#### 4.2.3. Standard Solutions

The TH and CA control standards (0.5 mg), respectively, were weighed precisely and dissolved in 1.0 mL of HPLC grade methanol. Using a Millipore membrane (0.22 μm), the supernatant was filtered.

#### 4.2.4. Mass Spectrum Condition

An ExionL C coupled to a SCIEX Triple TOF 5600 + (AB Sciex, Concord, ON, Canada) mass spectrometer was used to analyze the MT composition; mobile phases: acetonitrile (A) and 0.1% formic acid/water (B). An ACQUITY HSS T3 reversed-phase column (1.8 μm, 100 mm × 2.1 mm) was used for chromatography (Waters Corp., Milford, MA, USA). The method of gradient elution was: 0 min 5% B; 2 min 5% B; 14 min 98% B; 17 min 98% B; 17.1 min 5% B; and 20 min 5%, with a 0.3 mL/min flow rate. The column temperature was 30 °C; the quantity of the injections was 1.0 μL; the mass spectrum evaluation used an ESI source; and the sample was detected in both the positive and negative ion mode, with an MS1-MS2 mass scanning range of *m*/*z* 100 to 1200. The temperature of the ion source was 100 °C, and the desorption temperature was 500 °C. The number of positive and negative primary reiterations is 1, and the ion accumulation time is 30 ms; the number of positive and negative secondary reiterations is 12, the collision energy range is theoretical frequency ±20, and the ion accumulation time is 50 ms; the collision energy was −40 ne V.

#### 4.2.5. Animal Experiment Design and Drug Treatments

A total of 50 specific pathogen-free (SPF) grade ICR mice (male, 8 weeks old), weighing 20–25 g, were obtained from Beijing Vitahe Experimental Animal Technology Co., Ltd. (Beijing, China) and kept at the Experimental Animal Research Center of Minzu University of China. The laboratory temperature was 23 ± 1 °C, the relative humidity was 50 ± 10%, and a 12 h light/dark cycle was used for 1 week to acclimatize the mice. All animals was provided with free access to water and food during the acclimatization period. 

Following 7 days of acclimatization and feeding, 50 mice were randomly divided into 5 sets, with 10 mice in each group. The specific grouping settings are as follows: (1) normal control group (normal group); (2) Cis-model group (Cis group); (3) positive group (Cis+Amifostine group); (4) low-dose MT group (Cis+L-MT group); and (5) high-dose MT group (Cis+H-MT group). MT was dispersed in 5% HP-β-CD solution, and the mice in the MT low/high group were given 1.65 g/kg and 3.31 g/kg MT extract orally by gavage (calculated as 2.5 times and 5 times the clinical dose administered), respectively. The normal control group, the model group, and the positive group were provided with the identical volume of soluble vector. Gavage was administered continuously for 10 days. On day 7, 0.5 h after administration, the positive group was pretreated with amifostine (400 mg/kg, i.p), and 1 h after gavage, Cis (20 mg/kg, i.p) was injected into all mice, except for those in the normal control group, to induce AKI. After 3 days, all mice were anesthetized with isoflurane gas. After dissection, the nephron tissues were excised from the mice, washed with ice-cold saline, blotted dry, weighed, and the kidney index (kidney weight/body weight) was calculated. The right kidney tissue was rapidly refrigerated in liquid nitrogen and held in a refrigerator at -80 °C for obtaining the biochemical markers, and the left kidney tissue was immersed in a 4% paraformaldehyde solution for histopathological analysis.

#### 4.2.6. Serum Biochemical Analysis

Finally, the mice were subjected to a 24 h fast. Blood was taken from the orbital venous plexus of the mice, and the blood was centrifuged twice at 3500 r/min at 4 °C to obtain supernatants for biochemical analysis. CRE and BUN activity were assayed, according to the instructions of the centrifuge device manufacturer.

#### 4.2.7. Histopathological Examination

The renal tissue immobilized in 4% paraformaldehyde solution was cut into 5 μm slices. All sections were dewaxed to water with graded ethanol and dyed using H&E solvent and PAS reagent. Using a light microscope (Flexstation 3, Molecular Devices, Silicon Valley, CA, USA), histopathological changes were observed and photographed. Image analysis was performed using Image Pro Plus 6.0 image analysis software. The scoring criteria for the HE sections were as follows: normal renal tubules were scored as 0 points; 1–15% tubular damage was scored as 1 point; 15–25% tubular damage was scored as 2 points; 25–50% tubular damage was scored as 3 points; 50–75% tubular damage was scored as 4 points; and more than 76% tubular damage was scored as 5 points. In each case, 10 glomeruli were randomly observed under high magnification (×400), and the percentage of the relative area of the glomerular tunica and basement membrane was calculated (area of positive PAS staining in the glomerulus/total area of the glomerulus in cross-section ×100%).

#### 4.2.8. Assay of Antioxidant Enzyme Vitality and MDA Levels

The day after obtaining the kidney, all antioxidant enzymes were measured. Small pieces of kidney tissue were homogenized with ice saline (1:9, w:v) using a tissue grinder at low temperature to acquire a 10% kidney tissue homogenate. The temperature was 4 °C, the sample was centrifuged at 3500 rpm for 15 min, and the homogenate was centrifuged to obtain the supernatant. Changes in the indicators in the nephron homogenates were measured with an enzyme marker (Flexstation 3, Molecular Devices, Silicon Valley, CA, USA) using CAT, MDA, SOD, T-AOC, and GSH-Px kits, following the manufacturer’s instructions and the method which was outlined by Xu et al. [55].

#### 4.2.9. Quantitative Real-Time PCR Analysis

After obtaining an appropriate amount of kidney tissue for homogenization, RNA was derived using the Trizol reagent method, and a nanodropper (Allsheng, Hangzhou, China) was used to assay the concentration of RNA and the ratio of optical density of RNA. The RNA was retrotranscribed to cDNA using Star script II RT Mix with a gDNA Remove kit (GenStar, Beijing, China). The q RT-PCR was completed on a Light Cycler 96 system (Roche, Atlanta, GA, USA) to test the expressed levels of the selected inflammatory factors. The selected factors and primer sequences are shown in Table 3. The web-based tool https://primer3.ut.ee/, accessed on 14 March 2022) was used to design these primers, based on published sequences. Primer sequence similarity to other known sequences was checked with BLAST (www.ncbi.nlm.nih.gov/blast/Blast.cgi, accessed on 14 March 2022).

#### 4.2.10. Western Blot Analysis

The potential signaling pathways implicated in the renoprotective effects of MT were analyzed, and the expressed levels of related proteins were determined. Renal tissues were removed from -80 °C, cut into small pieces, and lysed in radio-immuno-precipitation assay (RIPA) lysis buffer, which was used for determining the total protein concentration by employing the BCA kit. Each group of proteins was separated by SDS-polyacrylamide gel electrophoresis (SDS-PAGE) and moved to polyvinylidene difluoride membranes (PVDF, Millipore, Burlington, MA, USA). The PVDF were held in 3% BSA for 60 min and then soaked for one night with primary antibodies against p65, p--p65, Nrf2, β-actin, HO-1, NQO1, Bax, p-IKKβ, IKKβ, Bcl-2, caspase 3, and Cleaved caspase 3 at 4 °C. Subsequently, the PVDF was incubated with the corresponding secondary antibodies for 120 min at 24 ± 2 °C. Protein expression was detected in a Multifunctional Imaging Analysis System (Azure Biosystems, Dublin, CA, USA), with an ECL chemiluminescence assay.

#### 4.2.11. Statistical Analysis

All data in this paper were statistically resolved using SPSS 25.0 (IBM SPSS Statistics, USA). All data are calculated as the mean ± standard deviation. Variations between each group of data were subjected to one-way ANOVA, employing LSD as a post hoc test, and *p* < 0.05 was regarded as statistically meaningful.

## 5. Conclusions

Taken together, this report suggests that MT may ameliorate Cis-induced renal injury by reducing oxidative stress, inflammation, and apoptosis. It was demonstrated that MT can improve renal function and has practical potential for clinical therapeutic application.

## Figures and Tables

**Table 1:** The identification of 39 components in MT using UPLC-Q/TOF-MS.

No.	RT (min)	Name	Formula	Ion.	Cal. *m*/*z*	Mea. *m*/*z*	Error (ppm)	MS/MS
1	2.70	Gallic acid	C_7_H_6_O_5_	M − H	169.01425	169.01445	1.2	169.0156, 125.0241, 97.0292, 69.0349
2	3.05	Protocatechuic acid	C_7_H_6_O_4_	M − H	153.01933	153.01959	1.7	153.0198, 109.0291, 108.0215, 91.0187
3	3.22	Chlorogenic acid	C_16_H_18_O_9_	M − H	353.08781	353.08801	0.6	191.0559, 179.0349, 161.0249, 135.0444
4	3.22	Shikimic acid	C_7_H_10_O_5_	M − H	173.04555	173.04599	2.6	173.0469, 109.0280, 93.0341
5	3.56	Esculetin	C_9_H_6_O_4_	M − H	177.01933	177.01944	0.6	177.0189, 149.0241, 133.0294, 77.0399, 105.0501, 89.0388
6	3.58	Caffeic acid	C_9_H_8_O_4_	M − H	179.03498	179.0351	0.6	135.0448, 134.0374, 89.0389
7	3.66	Vanillic acid	C_8_H_8_O_4_	M − H	167.03498	167.03526	1.7	167.0392, 152.0129, 123.0469, 108.0231, 91.0183
8	3.71	3-*O*-Feruloyl-quinic acid	C_17_H_20_O_9_	M − H	367.10346	367.10322	−0.7	191.0563, 173.0456, 155.0358, 134.0373, 93.0346, 67.0193
9	4.25	Rutin	C_27_H_30_O_16_	M − H	609.14611	609.14622	0.2	609.1476, 301.0367, 300.0284, 271.0267, 151.0033
10	4.25	Scopoletin	C_10_H_8_O_4_	M + H	193.04954	193.04957	0.2	178.0255, 149.0585, 133.0282, 122.0359
11	4.30	3′,5′-Dimethoxy-4′-hydroxyacetophenone	C_10_H_12_O_4_	M − H	195.06628	195.06683		195.0670, 136.0530, 119.0506
12	4.47	Isoferulic acid	C_10_H_10_O_4_	M − H	193.05063	193.05066	0.2	193.0504, 178.0255, 149.0248, 134.0372, 133.0289
13	4.82	Isochlorogenic acid A	C_25_H_24_O_12_	M − H	515.1195	515.11967	0.3	353.0870, 191.0555, 179.0343, 135.0443
14	5.51	4-Hydroxybenzoic acid	C_7_H_6_O_3_	M − H	137.02442	137.02438	−0.3	93.0347, 65.0397
15	5.95	Tenacigenin B	C_21_H_32_O_5_	M − H	363.2177	363.21777	0.2	327.1965, 311.1659, 276.1371,
16	6.20	Xanthyletin	C_14_H_12_O_3_	M − H	227.07137	227.07162	1.1	212.0469, 199.0771, 183.0448, 155.0496
17	6.55	Quercetin	C_15_H_10_O_7_	M − H	301.03538	301.03508	−1	301.0353, 178.9999, 151.0041
18	6.60	Wogonin	C_16_H_12_O_5_	M + H	285.07575	285.07628	1.9	285.0753, 270.0522, 253.0493, 213.0542, 197.0600
19	6.67	Kaempferol	C_15_H_10_O_6_	M − H	285.04046	285.04078	1.1	285.0412, 175.0402, 151.0045, 133.0229
20	6.74	Acacetin	C_16_H_12_O_5_	M − H	283.0612	283.06135	0.5	283.0605, 268.0367, 224.0468, 195.0460, 167.0495, 132.0206
21	7.7	Apigenin	C_15_H_10_O_5_	M − H	269.04555	269.04566	0.4	269.0473, 151.0033, 117.0347
22	8.34	Isoliquiritigenin	C_15_H_12_O_4_	M + H	257.08084	257.08059	−0.9	257.0806, 147.0422, 137.0232, 119.0493
23	8.36	Liquiritigenin	C_15_H_12_O_4_	M − H	255.06628	255.06634	0.2	255.0661, 135.0081, 119.0495
24	8.61	3-*O*-β-d-glucopyranosyl-(1→4)-6-deoxy3-*O*-methyl-β-d-allopyranosyl-(1→4)-β-d-oleandro-pyranosyl-11α-*O*-acetyltenacigenin B	C_43_H_68_O_18_	M − H	871.43329	871.43215	−1.3	811.4124, 829.4221, 667.3720, 631.3589
25	8.97	12-*O*-tigloyltenacigenin A	C_26_H_38_O_6_	M + H	447.27412	447.27306	−2.4	347.2206, 329.2100, 311.2002, 293.1893
26	9.61	3-*O*-β-d-glucopyranosyl-(1→4)-6-deoxy3-*O*-methyl-β-d-allopyranosyl-(1→4)-β-d-oleandro-pyranosyl-11α-*O*-Tigloyl-12β-*O*-acetyltenacigenin C	C_48_H_76_O_20_	M − H	971.48572	971.48517	−0.6	811.4116, 775.3915, 613.3334
27	10.94	Glycyrrhizic acid	C_42_H_62_O_16_	M + H	823.41106	823.40643	−5.6	647.3764, 471.3443
28	12.32	marsdenoside D	C_40_H_64_O_13_	M − H	751.42742	751.42489	−3.4	751.4214, 667.3692
29	13.05	Tenacissoside G	C_42_H_64_O_14_	M + H	793.43688	793.43266	−5.3	639.3783, 651.3702, 633.3592, 347.2195, 311.1994
30	13.55	11α,12β-Di-*O*-tigloyltenacigeninB	C_31_H_44_O_7_	M − H	529.31598	529.3138	−4.1	347.2208, 329.2102, 311.1993, 293.1898, 203.1068
31	13.56	Isokobusone	C_14_H_22_O_2_	M − H	221.1547	221.15386	−3.8	205.1239, 141.8689
32	13.65	Tenacissoside H	C_42_H_66_O_14_	M + H	795.45253	795.44815	−5.5	633.3608, 431.2743, 329.2097, 311.2020
33	13.82	11α-*O*-Tigloyl-12β-*O*-Benzoyltenacigenin B	C_33_H_42_O_7_	M + H	551.30033	551.29757	−5	433.2358, 329.2119, 311.2010, 293.1906
34	14.00	marstenacisside B5	C_57_H_90_O_24_	M − H	1157.57493	1157.57149	−3	1055.5049, 995.5205
35	14.25	11α-*O*-2-Methylbutyryl-12β-*O*-2-tigloyl tenacigeninB	C_31_H_46_O_7_	M + H	531.33163	531.32961	−3.8	329.2114, 311.2002, 293.1908, 203.1077
36	14.47	11α-*O*-2-Methylbutyryl-12β-*O*-2-benzoyl tenacigeninB	C_33_H_44_O_7_	M + H	553.31598	553.31245	−6.4	329.2104, 311.1989, 293.1882
37	15.99	Glycyrrhetinic acid	C_30_H_46_O_4_	M − H	469.33233	469.33067	−3.6	469.3301, 425.3414
38	16.41	Betulinic acid	C_30_H_48_O_3_	M + H	457.36762	457.36687	−1.6	457.3652, 161.1799
39	16.93	Oleanolic acid	C_30_H_48_O_3_	M − H	455.35307	455.3526	−1	455.3518

**Table 2:** Antibody source and dilution ratio.

Name	Company	Lot Number	Dilution Ratio
Anti-p65	Bioss, Beijing, China	Lot: BB10125523	1:1000
Anti-Bax	Bioss, Beijing, China	Lot: BA12063356	1:1000
Anti-Nrf2	Bioss, Beijing, China	Lot: BB01286971	1:1000
Anti-Bcl-2	Bioss, Beijing, China	Lot: BB09268767	1:1000
Anti-HO-1	Bioss, Beijing, China	Lot: BB07252595	1:1000
Anti-NQO1	Bioss, Beijing, China	Lot: BB10121621	1:1000
Anti-caspase 3	Bioss, Beijing, China	Lot: BA08247137	1:1000
Anti-cleaved caspase 3	Abcam, Cambridge, UK	Lot: ab2302	1:1000
Anti-p-p65	Abcam, Cambridge, UK	Lot: ab86299	1:1000
Anti--IKKβ	Abcam, Cambridge, UK	Lot: ab124957	1:1000
Anti-p-IKKB	Abcam, Cambridge, UK	Lot: ab59195	1:1000
Anti-β-actin	Servicebio, Wuhan, China	Lot: AC220730001	1:1000
Lamin B	Abcam, Cambridge, UK	Lot: ab0054	1:1000

**Table 3:** Primers used for RT-PCR analysis.

Gene	Primer Sequence (5′ to 3′)	Length	Accession Number
TNF-α	F: CAGGCGGTGCCTATGTCTC	19	NM_013693.3
	R: CGATCACCCCGAAGTTCAGTAG	22	
IL-1β	F: GCAACTGTTCCTGAACTCAACT	22	NM_008361.4
	R: ATCTTTTGGGGTCCGTCAACT	21	
IL-6	F: TAGTCCTTCCTACCCCAATTTCC	23	NM_031168.2
	R: TTGGTCCTTAGCCACTCCTTC	21	
β-actin	F: GGCTGTATTCCCCTCCATCG	20	NM_007393.1
	R: CCAGTTGGTAACAATGCCATGT	22	

## Data Availability

Data are contained within the article and Supplementary Materials.

## Supplementary Materials

The following supporting information can be downloaded at: https://www.mdpi.com/article/10.3390/molecules28227582/s1, Figure S1: Original Images for Blots and Gels.

## Abbreviations

MT	*Marsdenia tenacissima*	Cis	cisplatin
XAP	Xiaoaiping	p-IKKβ	phospho-Inhibitor of nuclear factor kappa B kinase beta subunit
AKI	acute kidney injury	IKKβ	inhibitor of nuclear factor kappa B kinase beta subunit
CRE	creatinine	Bax	Bcl-2-associated X
BUN	blood urea nitrogen	HO-1	heme oxygenase-1
HE	hematoxylin–eosin staining	NQO1	recombinant NADH dehydrogenase, quinone 1
PAS	periodic acid–Schiff staining	Bcl-2	B-cell lymphoma-2
Nrf2	nuclear factor-erythroid 2-related factor 2	ROS	reactive oxygen species
NF-κB	nuclear factor kappa-B	SOD	superoxide dismutase
IL-1β	interleukin-1β	GSH-Px	glutathione peroxidase
IL-6	interleukin-6	CAT	catalase
TNF α	tumor necrosis factor-α	TCM	traditional Chinese medicine
TH	tenacissoside H	T-AOC	total antioxidant capacity
p-p65	phospho-p65	MDA	malondialdehyde
CA	chlorogenic acid	SCR	serum creatinine
WB	Western blot	SPF	specific pathogen-free
RIPA	radioimmunoprecipitation assay	SDS-PAGE	SDS-polyacrylamide gel electrophoresis
PVDF	polyvinylidene fluoride	TIC	total ion chromatography
p65	NFκB p65 protein	HP-β-CD	2-hydroxypropyl-β-cyclodextrin
