# Supplementary material for: The Protective Effect of Marsdenia tenacissima against Cisplatin-Induced Nephrotoxicity Mediated by Inhibiting Oxidative Stress, Inflammation, and Apoptosis
Source: Molecules. 2023 Nov 14;28(22):7582. doi: 10.3390/molecules28227582 (PMC10674371; doi:10.3390/molecules28227582)
Supplement: Supplementary file 1 [file molecules-28-07582-s001.zip › molecules-2592338-supplementary.pdf]

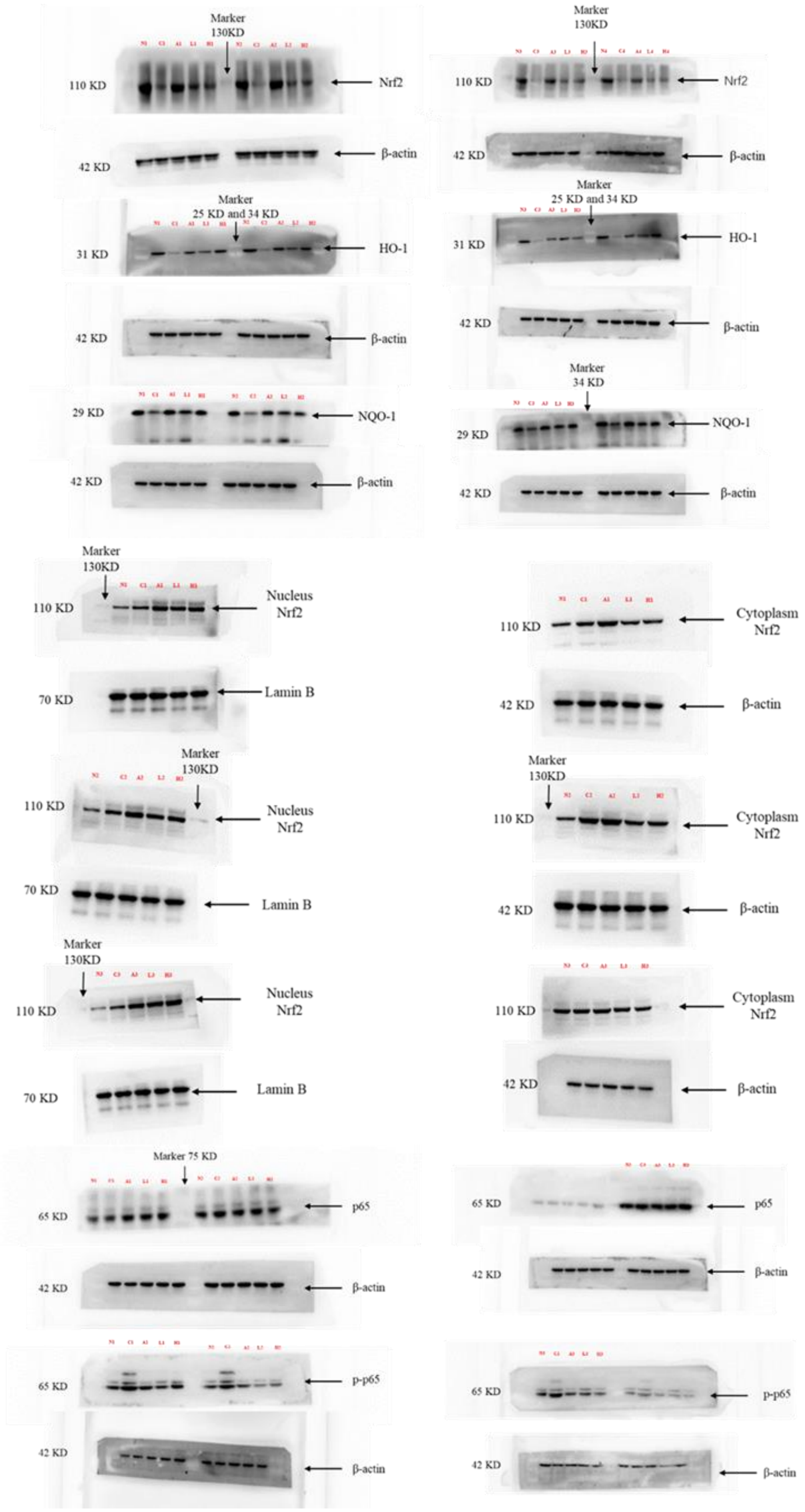

Marker
130KD
110 KD
Nrf2
42 KD
β-actin
Marker
130KD
110 KD
Nrf2
42 KD
β-actin
Marker
25 KD and 34 KD
31 KD
HO-1
42 KD
β-actin
Marker
25 KD and 34 KD
31 KD
HO-1
42 KD
β-actin
29 KD
NQO-1
42 KD
β-actin
Marker
34 KD
29 KD
NQO-1
42 KD
β-actin
Marker
130KD
110 KD
Nucleus
Nrf2
70 KD
Lamin B
110 KD
Cytoplasm
Nrf2
42 KD
β-actin
Marker
130KD
110 KD
Nucleus
Nrf2
70 KD
Lamin B
Marker
130KD
110 KD
Cytoplasm
Nrf2
42 KD
β-actin
Marker
130KD
110 KD
Nucleus
Nrf2
70 KD
Lamin B
110 KD
Cytoplasm
Nrf2
42 KD
β-actin
Marker 75 KD
65 KD
p65
42 KD
β-actin
65 KD
p65
42 KD
β-actin
65 KD
p-p65
42 KD
β-actin
65 KD
p-p65
42 KD
β-actin

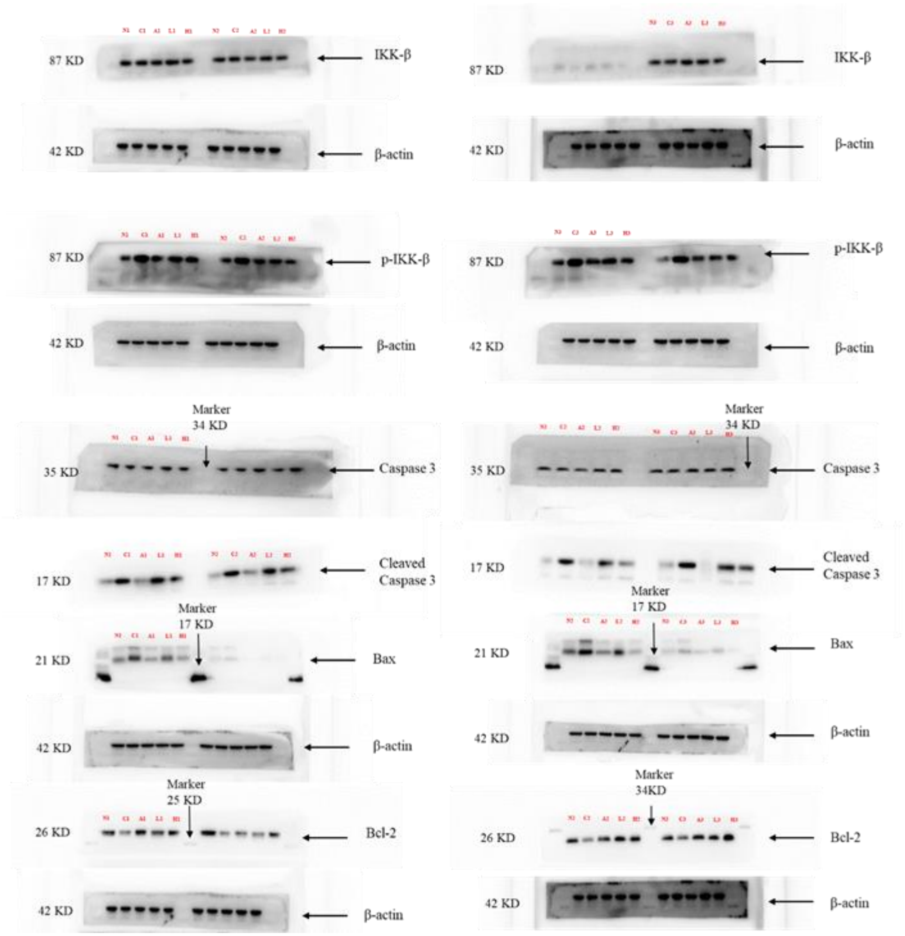

Figure S1. Original Images for Blots and Gels; Abbreviations: N means normal group; C means model group (Cis group); A means Amifostine group (positive drug group); L means low dose group; H means high dose group.
